# Reducing the Influence of Environmental Factors on Performance of a Diffusion-Based Personal Exposure Kit

**DOI:** 10.3390/s21144637

**Published:** 2021-07-06

**Authors:** Huixin Zong, Peter Brimblecombe, Li Sun, Peng Wei, Kin-Fai Ho, Qingli Zhang, Jing Cai, Haidong Kan, Mengyuan Chu, Wenwei Che, Alexis Kai-Hon Lau, Zhi Ning

**Affiliations:** 1 Division of Environment and Sustainability, The Hong Kong University of Science and Technology, Hong Kong, China; hzongab@connect.ust.hk (H.Z.); lsunaj@connect.ust.hk (L.S.); pweiaa@connect.ust.hk (P.W.); mchuaf@connect.ust.hk (M.C.); wenweiche@ust.hk (W.C.); alau@ust.hk (A.K.-H.L.); 2 Department of Marine Environment and Engineering, National Sun Yat-sen University, Kaohsiung 80424, Taiwan; p.brimblecombe@uea.ac.uk; 3 JC School of Public Health and Primary Care, The Chinese University of Hong Kong, Hong Kong, China; kfho@cuhk.edu.hk; 4 Key Laboratory of Public Health Safety, Ministry of Education, Fudan University, Shanghai 200433, China; 18111020018@fudan.edu.cn (Q.Z.); jingcai@fudan.edu.cn (J.C.); kanh@fudan.edu.cn (H.K.); 5 Department of Environmental Health, School of Public Health, Fudan University, Shanghai 200032, China

**Keywords:** air quality monitoring, baseline correction, electrochemical gas sensor, Hong Kong, microenvironments, personal exposure evaluation

## Abstract

Sensor technology has enabled the development of portable low-cost monitoring kits that might supplement many applications in conventional monitoring stations. Despite the sensitivity of electrochemical gas sensors to environmental change, they are increasingly important in monitoring polluted microenvironments. The performance of a compact diffusion-based Personal Exposure Kit (PEK) was assessed for real-time gaseous pollutant measurement (CO, O_3_, and NO_2_) under typical environmental conditions encountered in the subtropical city of Hong Kong. A dynamic baseline tracking method and a range of calibration protocols to address system performance were explored under practical scenarios to assess the performance of the PEK in reducing the impact of rapid changes in the ambient environment in personal exposure assessment applications. The results show that the accuracy and stability of the ppb level gas measurement is enhanced even in heterogeneous environments, thus avoiding the need for data post-processing with mathematical algorithms, such as multi-linear regression. This establishes the potential for use in personal exposure monitoring, which has been difficult in the past, and for reporting more accurate and reliable data in real-time to support personal exposure assessment and portable air quality monitoring applications.

## 1. Introduction

Assessments of exposure to air pollutants have often depended heavily on measurements from stationary reference instruments, but these may poorly represent individual exposure linked to the pattern of human activity. Conventional networks do not accurately represent concentrations in the microenvironments experienced by people. Such networks are costly, require considerable care in housing and maintenance and are not easily moved. They fail to provide data that reflect the high degree of spatial and temporal variation that contributes to personal exposure in urban microenvironments [1,2,3]. It is not practical to improve exposure assessment by adding conventional sites, so this study will evaluate the accuracy of a sensor-based portable monitoring unit, which includes a novel dynamic baseline tracking approach to deal with the effects of humidity and temperature on the observations made in assessing real world exposure. Such monitoring needs are driven by a global concern from governmental institutions, the research community and the general public over exposure to air pollution. Over 90% of the population worldwide is exposed to ambient pollution levels which exceed World Health Organization guidelines [4]. Numerous researchers have shown a consistent association between poor air quality and adverse human health outcomes [5], but such work typically uses pollution data from fixed-site outdoor monitoring networks for estimating mortality and morbidity [6,7,8].

In the last decade, the development of microsensor-based monitoring methods has shown potential to address many of the limitations in exposure estimation imposed by conventional monitoring networks. Sensor technology benefits from portability and low cost and can represent locations where individuals are exposed to air pollution [9,10,11]. In particular, it is applicable to dynamically changing environments, such as those found at the roadside, indoors and inside transit systems. Microsensors are also employed to provide data during long-term deployment into areas where conventional monitoring is not available or practical. However, measurements made with these sensors are affected by common environmental factors, especially temperature and humidity, as well as interference from other common urban pollutants. After initial calibration, data from these sensors commonly drifts over time, requiring care to account for such problems [12,13]. A critical issue for measurement in microenvironments arises from either transient peaks or diurnal time scale changes in temperature and humidity. A transient loss of calibration commonly occurs when monitors are moved between indoors and outdoors or between air conditioned and unconditioned spaces over periods of seconds or minutes. One of the most common types of gas sensors, based on electrochemical principles, shows both positive and negative interference from rapidly changing temperature and humidity encountered in an individual’s daily life, and poses special challenges for stable and reliable performance during exposure evaluation.

Improving sensor measurement is a key aspect for further research. Simple linear regression, which assumes the linearity of sensor responses and pollutant concentration from regular reference instruments, is not suitable for dynamic microenvironments with abrupt environmental variation. Mathematical algorithms to compensate for sensor response to environmental variation have used multi-linear regression [14,15] and artificial neural networks (ANN) [16]. Another approach is to apply baseline correction methods, where a baseline is extracted by determining the minimum measurement within a specific time interval with ambient temperature and humidity and allowing these to serve as explanatory variables over a range of meteorological conditions [11,17]. Such approaches have primarily been used with data from long-term, week- or month-long deployments of sensor-based systems under ambient conditions [18]. However, long-term algorithms may fail to account for sudden changes of microenvironments, often of interest in exposure studies [19,20].

This study assessed the performance of a sensor-based personal exposure kit (Model PEK-Standard, Sapiens, Hong Kong) in exposure applications. The kit directly outputs real-time pollutant concentrations, thus providing the ability to determine individual exposure. The concentration data from the PEK with its dynamic baseline tracking were compared with raw sensor output using commonly employed universal correction algorithms [15,21] often used to compensate for the combined effects of temperature and humidity variation. The work reported here evaluates the accuracy of the novel method in terms of output robustness and applicability in rapidly changing environments to assess its ability to meet the requirements of exposure studies.

## 2. Methodology

### 2.1. Description of the Personal Exposure Kit (PEK)

**Figure 1:**
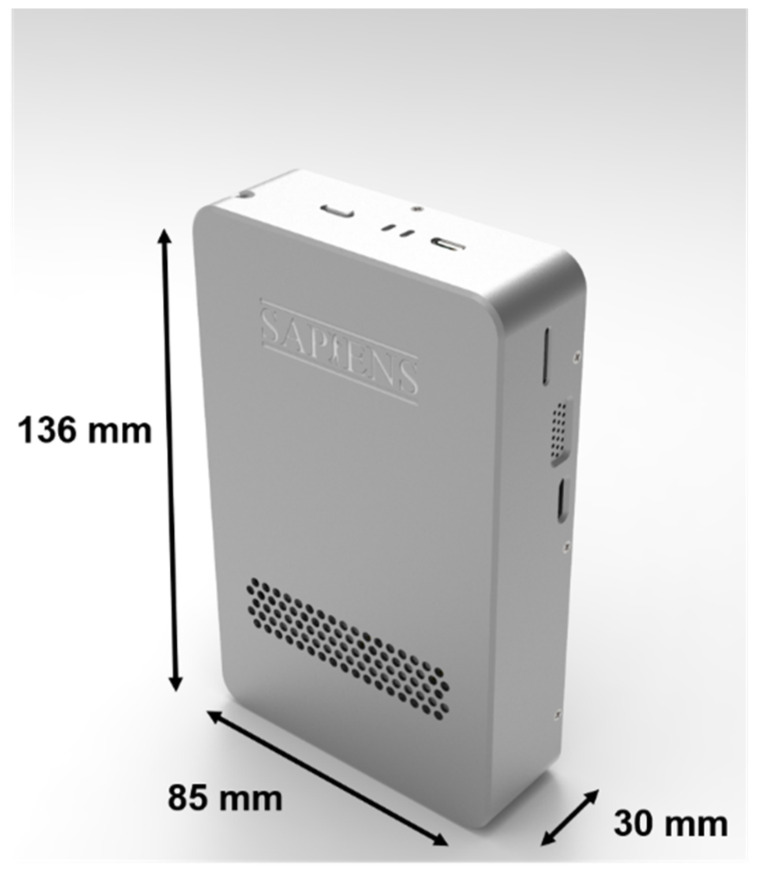
Personal Exposure Kit (PEK): The orthograph of the unit with dimensions of 136 mm × 85 mm × 30 mm.

The PEK (Figure 1) used for this study is a microsensor-based monitoring device designed for portable and continuous air pollution measurements and includes a dynamic baseline feature to enhance performance. It is compact (136 mm × 85 mm × 30 mm), lightweight (<500 g) and runs on an internal battery. Although the kit has sufficient battery capacity to run for three days, it can also be powered and recharged using a 12-volt supply. The device employs passive diffusion-based sampling and operates noiselessly. The PEK model used here directly measures the concentration of CO, NO_2_ and O_3_ simultaneously. Each gas is measured by a separate gas module based on electrochemical sensing and pair differential filter (PDF) technology (PCT patent pending) with real-time dual signal outputs of simultaneous *raw* signal and dynamic *baseline* signal for the individual gas sensors. The two signal outputs in volts, together with concentration outputs from the PEK in parts per billion (ppb), are examined here to determine the effectiveness of the method in reducing sensitivity to humidity and temperature. The sensor modules are comprised of raw sensor heads of A-Type 4-electrode electrochemical sensors from Alphasense (CO-A4, NO_2_-A43F, and Ox-A431) to provide the *raw* signal and corresponding proprietary baseline sensor heads to provide the *baseline* signal. The Ox-A431 sensor responds to oxidizing gases (principally O_3_ and NO_2_ in ambient air), while the NO_2_ sensor includes a filter that removes O_3_ such that the sensor responds to NO_2_, the difference giving an estimate of the O_3_ concentration. Temperature, humidity (SHT20, Sensirion, Switzerland) and light intensity (BH1750FVI, Rohm, Japan) are measured simultaneously to provide the environmental conditions to differentiate the microenvironments [22]. The default sampling resolution is 1 min and real-time sensor signal data is continuously and automatically measured by PEK and the on-board microprocessor provides an output of real-time pollutant concentration after processing the raw data. The raw data and concentration data are stored on a removable SD card and simultaneously transmitted to a cloud server by 4G module. The PEK is also equipped with a calibration manifold kit made of Teflon to fit the gas sensor diffusion path and provide a sealed flow-through for calibration using gas standards.

### 2.2. Laboratory Tests

Sensor system performance traits were evaluated experimentally in a range of laboratory environments.

#### 2.2.1. Laboratory-Based Testing Protocols

The laboratory setup consisted of several subcomponents: real-time data acquisition, an environmentally controlled chamber (LK-150G, Kingjo Ltd., Dongguan, China), and the kit which was placed inside the chamber. The flow-through calibration allowed concentrations of gases in the air to be set using a standard gas supply, diluted to the required concentration. Target CO standard gas concentrations were produced with a dynamic calibrator (T700U, Teledyne, Sauzend Oaks, CA, USA), which combined zero-gas generated using a zero-air generator (701, Teledyne, Sauzend Oaks, CA, USA) with compressed CO from a cylinder (100 ppm CO, Linde HKO Ltd., Hong Kong, China). NO_2_ and O_3_ were generated with a NO_2_/NO/O_3_ calibration source (714, 2B Technology, Boulder, CO, USA). During calibration, the gas flow rate was fixed at 3 L min^−1^ in all cases (CO, NO_2_ and O_3_), and validated with a flow meter (Defender 520, Mesa Labs, Lakewood, CO, USA). Prior to the experiments, both the T700U and 2B 714 instruments were left to warm up for 30 min. In all cases, the kit was allowed to warm up for 48 h to assure a steady-state response. Temperature and humidity measurements from the chamber were refined beyond the standard configuration by including a Vaisala Temperature/Humidity probe (HMP3, Vaisala HUMICAP, Helsinki, Finland). Data output was stored on an internal SD card during the experimental procedure. 

#### 2.2.2. Signal Linearity Test

It is important that gas sensors respond linearly over the concentration range of interest. The PEK sensor signals were recorded at a range of specific concentrations of the analyte gases (T = 22 °C, RH = 55%) during calibration procedures. In this study, standard gas concentrations were 50 ppb, 100 ppb, 200 ppb for NO_2_, as well as for O_3_; 0.2 ppm, 0.5 ppm, 1 ppm, 1.5 ppm and 2 ppm for CO. Stable values, at each test concentration, were maintained for 15 min. The PEK was placed in the calibration manifold kit connected to the gas supply with Teflon tubing. The tests were run one gas at a time, and to avoid interference NO_2_ was completed first followed by O_3_. 

#### 2.2.3. Effects of Environmental Factors on Sensor Baseline

One of the concerns in gas monitoring using electrochemical sensors is the effect of environmental factors on the sensor baseline, humidity in particular [21]. O_3_ sensor output is also influenced by the environment [14], especially rapid changes in humidity. The CO sensor shows the effects of diurnal temperature variation patterns [9], suggesting a strong interrelation between temperature and sensor output. Our work aimed to ensure the robustness of the sensor signal (V) in comparison with both the PEK output signal and raw sensor signal in the chamber under varying test conditions to ensure effective calibration across changing ambient conditions (T: 5 °C–40 °C, RH: 5–90% in 8 h and 20 min). 

#### 2.2.4. Sensor Response to Transient Pollutant Variation

Response time, typically as t_90_ or t_50_, is a performance characteristic of electrochemical sensors and measurement criterion [23]. The t_90_ is defined as the time taken for the sensor response signal to reach 90% of its steady state value; similarly, the t_50_ time refers to the time to reach 50% of the value. Two Nafion tubes (ME-110-24COMP4, PERMA PURE, Lakewood, NJ, USA), each 60 cm long, were connected in parallel to the gas delivery tubing, located inside the chamber to ensure a controlled environment [18]. This tubing allows passage of vapor phase water through its walls while retaining the pollutant gas. The kit was exposed to the target gases at a flow rate of 3 L min^−1^. The concentrations used were zero air or 2 ppm, 500 ppb and 200 ppb corresponding to CO, NO_2_ and O_3_. Sensor response times and concentration measurements were evaluated at constant temperature and humidity once a stable environment was established in the chamber (T = 22 °C, RH = 56%). 

#### 2.2.5. Sensor Response to Transient Variation of Temperature and Humidity

Another key performance requirement is sensor response to rapid changes of temperature and humidity, which can distort the concentration measurements during real world exposure. Examples include entering and exiting air conditioning, heated spaces or transit systems. Two separate experiments explored PEK operation under sudden changes in temperature and humidity. The variable temperature scenario (T) used a decrease from 40 °C to 15 °C over an hour. For humidity variation, it was arbitrarily fixed at 47%, then varied from 65% to 15% for 7 min at a constant temperature of 25 °C. 

### 2.3. Field Performance

Although temperature and humidity variation can be effectively controlled in the laboratory, the ambient environment is more complex with different factors affecting sensor response. The study also included intensive field performance assessment. The PEK was deployed at three different government monitoring sites in Hong Kong: Tseung Kwan O (TKO, a community station); Mong Kok (MK, a roadside station) and Kwun Tong (KT, a community station). The units were mounted on the railing on the opposite side of the road from the MK site, at the same height of its sampling inlet. All data from the conventional monitoring instruments as well as the PEK values were recorded at 1-min intervals. 

#### 2.3.1. Performance after Sensor Relocation

Sensor-based monitors used in the field are typically calibrated in the laboratory, using standard gases to establish basic sensor response, or via co-location with regular monitoring sites before and/or during use [24]. However, one troubling observation from prolonged field deployment is that a monitor may show calibration drift over time. This drift may be due to electronic or chemical changes in the sensors while in use. We adopted protocols to account for this:

- Laboratory calibration—to assess suitability for field use, a laboratory calibrated PEK was deployed at reference sites to evaluate the robustness of the calibration with respect to accuracy and precision.
- Field co-location calibration—to quantify sensor changes among different environments, calibration took place via co-location at the TKO site and was subsequently validated via co-location at the other two sites for three days.

#### 2.3.2. Performance in Dynamic and Changing Microenvironments

Robustness of the measurements across dynamic and changing microenvironments was established by simulating daily routines to reflect typical individual exposure profiles. Initially, the PEK was co-located with a well characterized and calibrated mini air station (MAS-AF300, Sapiens, Hong Kong, China) at Hong Kong University of Science and Technology which served as an indoor reference. Co-location initially took place from 06:00 to 08:00, as described in previous studies [10,18]. The kit was then carried at ~0.85 m height during this period and moved: (i) indoors (air-conditioned spaces), (ii) to reference sites and (iii) to outdoor sites, simulating a personal exposure profile. Two reference sites (TKO and MK) were chosen to provide data of known quality during hour-long visits to outdoor environments. 

### 2.4. Data Analysis

The PEK has three separate outputs: (i) a raw sensor output in volts as *Raw*, (ii) output in volts tuned by the dynamic baseline as *Baseline*, (iii) concentration output in ppb (hereafter PEK measurement). The algorithm used in the PEK to convert from sensor signals to concentration is as follows and the calculation is performed onboard of PEK for real-time concentration output.

Concentration = a × (Raw − Baseline) + b

Multi-linear regression models are commonly used in sensor adjustment algorithms. Previous researchers [14,25,26] have employed them to compensate for environmental factors, typically representing pollutant concentration (c) as:

Concentration = (*a*_1_ *RH* + *a*_2_*T* + *a*_3_) Δ*V* + *a*_4_ *RH* + *a*_5_ *T* + *b*

A widely used approach for mathematical baseline correction with a 4-electrode sensor subtracts the working electrode output from the reference electrode output and yields a differential voltage Δ*V*, which is equivalent to the raw signal output from the PEK. RH and T of the sampled air are measured directly from the PEK. The parameters *a*_1_, *a*_2_… are regression coefficients and *b* the intercept [14]. Data were processed with open-source packages including pandas, NumPy, and Matplotlib for data analysis and visualization [27].

## 3. Results and Discussion

### 3.1. Laboratory Tests

#### 3.1.1. Signal Linearity

**Figure 2:**
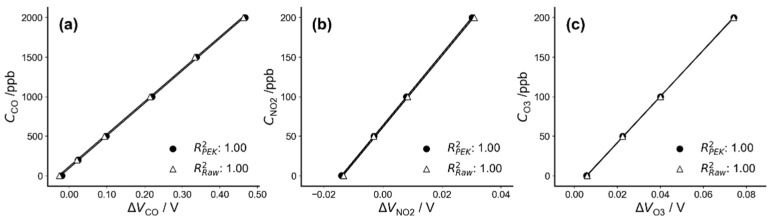
Correlation coefficients of baseline-corrected output as a function of raw sensor output for (**a**) CO, (**b**) NO_2_ and (**c**) O_3_ under controlled laboratory conditions (T = 22 °C, RH = 55%).

Figure 2 demonstrates the correlation between the PEK outputs as a function of the concentrations of target pollutants, including two separate voltage outputs, raw sensor output and corrected output from the PEK. The three pollutants (CO, NO_2_ and O_3_) show strong linear relationships over the calibration range, indicating the reliability of both sensor outputs under laboratory conditions, which is consistent with other results [9,12,14]. This also demonstrates that data correction does not unduly affect baseline-corrected output against raw sensor output in stable laboratory conditions.

#### 3.1.2. Analysis of Environmental Effects on Sensor Baseline

**Figure 3:**
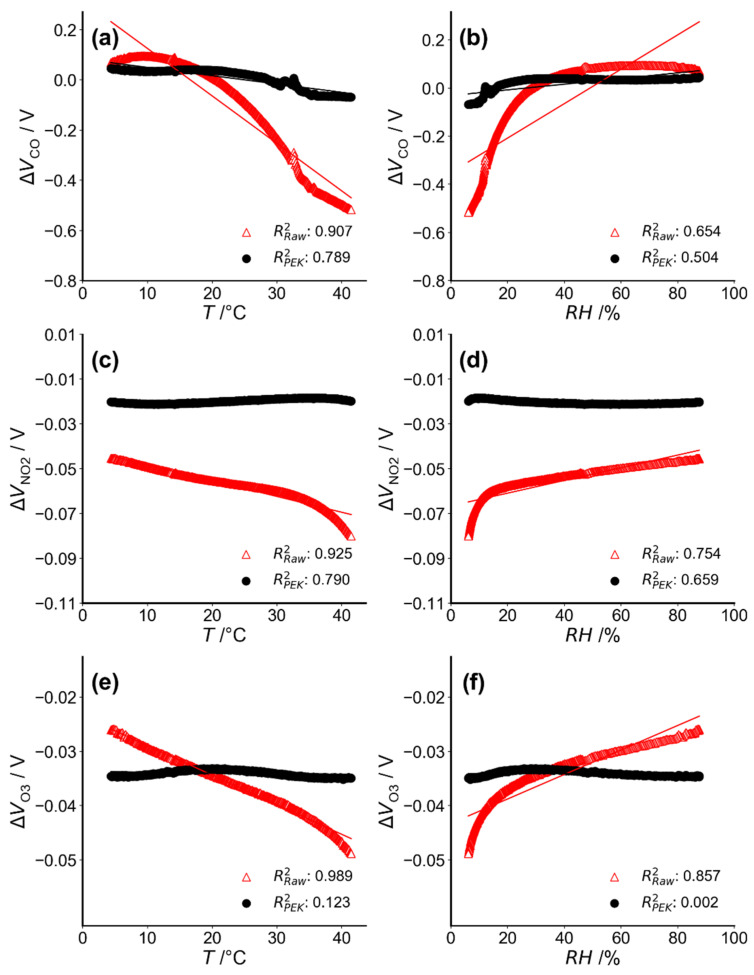
Raw sensor (red lines) and corrected output (black lines) responses to zero air against changing temperature (**a**,**c**,**e**) and humidity (**b**,**d**,**f**). Note: Corrected outputs are corrected using the dynamic baseline method.

Sensor output (corrected output and raw sensor output) from the environmental chamber are shown in Figure 3 over the temperature range 5 °C–40 °C (Figure 3a,c,e) and humidity between 5% and 90% (Figure 3b,d,f). These reflect conditions found in most indoor and outdoor environments. As expected, the raw uncalibrated output varies with environmental conditions. It correlates with temperature (*R*^2^ > 0.90) and with humidity (*R*^2^ > 0.65) for the three gas sensors. Temperature increases lead to a decrease in sensor voltage output and the output under RH increase shows a step-like character followed by an increase in voltage. The sensor voltage outputs for NO_2_ and O_3_ are more easily affected by environmental variables under high temperatures (T > 35 °C) or low humidity (RH < 20%) with piecewise linearity over the entire range (Figure 3c–e). The raw data is corrected in the PEK output where changes with temperature and humidity are much reduced, as indicated by linear regression slopes approaching zero. The corrections using the multiple regression equation are necessarily linear, and not perfect given the non-linear features seen in the raw output of Figure 3. However, the multiple regression used seems sufficient to correct the PEK output such that the dynamic baseline tracking technology reduces the effect of real-world changes.

#### 3.1.3. Response to Concentration and Simulated Ambient Conditions

**Figure 4:**
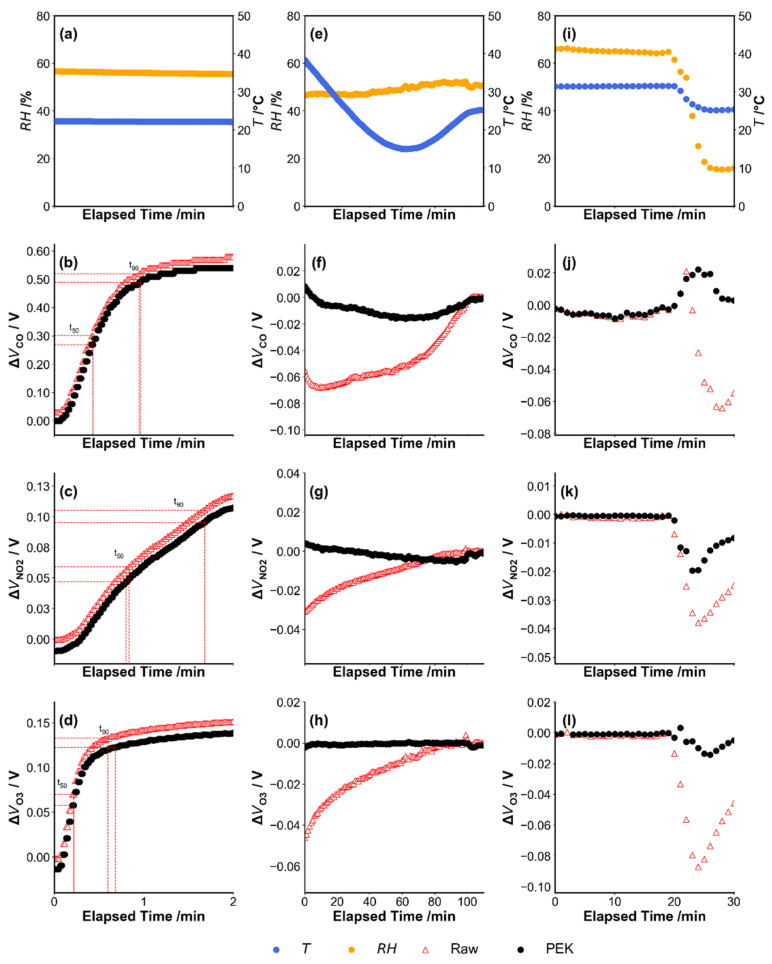
The profile of sensor response after switching the incoming air from zero air to pollutant gas until they reach steady state (**a**–**d**), and the profile of sensor response after the temperature of the environmental-controlled chamber changed from 40 °C to 15 °C while exposure to zero air (**e**–**h**), and the profile of sensor response when the zero air in the environmental-controlled chamber with stable climatic condition (T = 30 °C, RH = 65%) experienced a rapid change of relative humidity from 65% to 15% within 7 min (**i**–**l**).

Figure 4a–d shows the raw sensor output at fixed concentrations of standard gases when temperature and relative humidity are constant. For CO and O_3_, the t_90_ values were less than a minute at a flow of 3 L min^−1^ according to laboratory test (CO_Raw_ ~0.85 min, CO_PEK_ ~0.85 min, O_3,Raw_ ~0.6 min, O_3,PEK_ ~0.7 min), while the NO_2_ sensor took just a little longer to achieve stability (~1.5 min) due to the ozone absorbent filter on top of the NO_2_ sensor head that slightly reduces the air diffusivity. There was a slight time lag observed between the raw sensor output and corrected output for O_3_ and less being observed for CO and NO_2_. The lag may be due to the fact that the raw and corrected outputs come from two independent signals of raw and dynamic baseline tracked sensor heads with inherent difference of gas permeability from the surface to the electrode of individual sensors. However, the lag appears to be insignificant and no obvious discrepancies in data were observed for the three gases. Figure 4e–h shows that the raw sensor output and corrected output on exposure to zero air during a rapid change of temperature. The corrected output reduces the influence of changing temperature giving a nearly flat curve across the temperature range, while the raw sensor output shows a marked change before returning to a stable status once the temperature stabilizes. It is noted that the sensor raw signal profiles in response to the change of temperature and humidity have no clear linear relations as shown in the figures, and the inherent characteristics may limit the MLR method’s capability in correcting for the non-linear responses, compared with the physical and dynamic baseline tracking method for the correction. Figure 4i–l shows the changes of the raw sensor signals brought by the sudden variation of humidity and temperature. There was an immediate and remarkable deviation of sensor raw signal from the baseline at the inflection point of temperature and humidity profiles, similar to the observations in the literature [13]; however, the PEK sensor signal with dynamic baseline correction showed an effective suppression performance of signal deviation. Both the amplitude of signal perturbance and the time to recover from the condition change was greatly reduced as shown in the figure for all three types of gas sensors. The test was conducted in a simulated condition with sudden environment change, and the gradient change was expected to be less frequently encountered in real-world. Future studies will still be needed to quantify the relation of the temperature or humidity gradient and the PEK sensor signal response to further improve performance.

The PEK output incompletely reduces this impact when humidity reduces 50% in 9 min. However, CO and O_3_ PEK output signals recover from this humidity shock to the almost stable original state, while the raw sensor signal recovers more slowly under the same conditions. For NO_2_, such a transient humidity change affects both sensor signals, with the NO_2_ sensor data from the PEK shows a smaller effect and recovers more quickly than the raw sensor signal. The PEK returns to stability within ~5 min following a RH drop. Overall, these results show that output adjusted using dynamic baseline correction can reduce the effect of rapid changes in temperature and humidity on signal output, as is evident in the effects on raw sensor output.

Note: The elapsed time refers to the time period counted from the beginning of the experiment and the zero-point shown in the x-axis refers to the experiment starting point. The experiment in Figure 4i–l had air temperature varying in a narrow range between 25 °C and 30 °C, due to the limitation of the environmental-controlled chamber, and it was difficult to maintain constant temperature when humidity had a rapid change but the temperature range was relatively narrow.

### 3.2. Field Test Validation

#### 3.2.1. Applicability of Laboratory Calibration to Field Measurements

**Figure 5:**
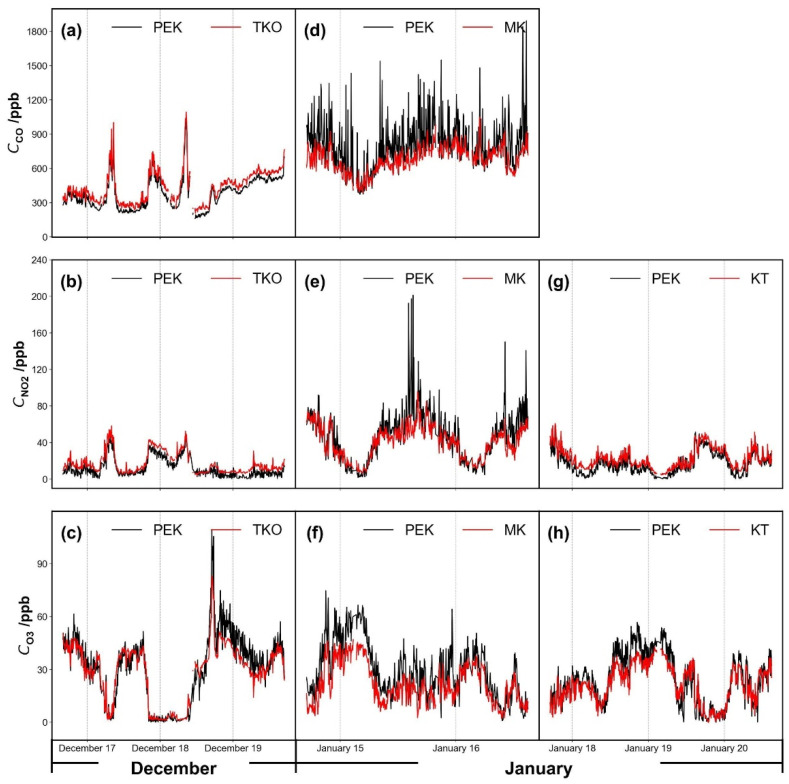
Time series plots for gases measured by the Personal Exposure Kit (PEK), calibrated under laboratory conditions (Methodology 2.3.1 (I)) as validated at the (**a**–**c**) Tseung Kwan O (TKO), (**d**–**f**) Mong Kok (MK) and (**g**,**h**) Kwun Tong (KT) air quality monitoring sites. Note: CO for Kwun Tong site was not available. Data resolution is set as 5 min.

At the MK roadside station, a large amount of sensor noise seems to arise from wind variation and local vehicle emissions (Figure 5). The underlined information measured by the PEK can match with the general trend of reference concentration variations. The bias and fluctuations derived from roadside traffic were corrected applying the spline minimum method [28,29] to both reference data and PEK measurements. This reduces local vehicle plume effects across discrete one-hour time windows, thus locating the 5% percentile data point in each time window. 

**Figure 6:**
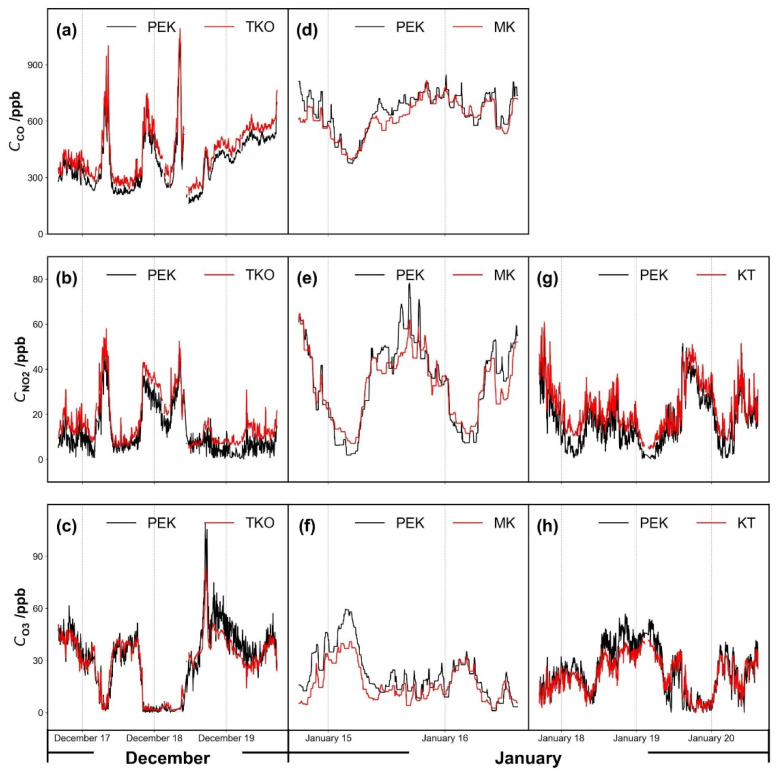
Pollutant concentrations corresponding to Figure 5: (**a**–**c**) Tseung Kwan O (TKO), (**d**–**f**) Mong Kok (MK) and (**g**,**h**) Kwun Tong (KT) air quality monitoring sites, except for Mong Kok (MK, **d**–**f**) which is corrected using the spline minimum method. Data resolution is set as 5 min.

**Figure 7:**
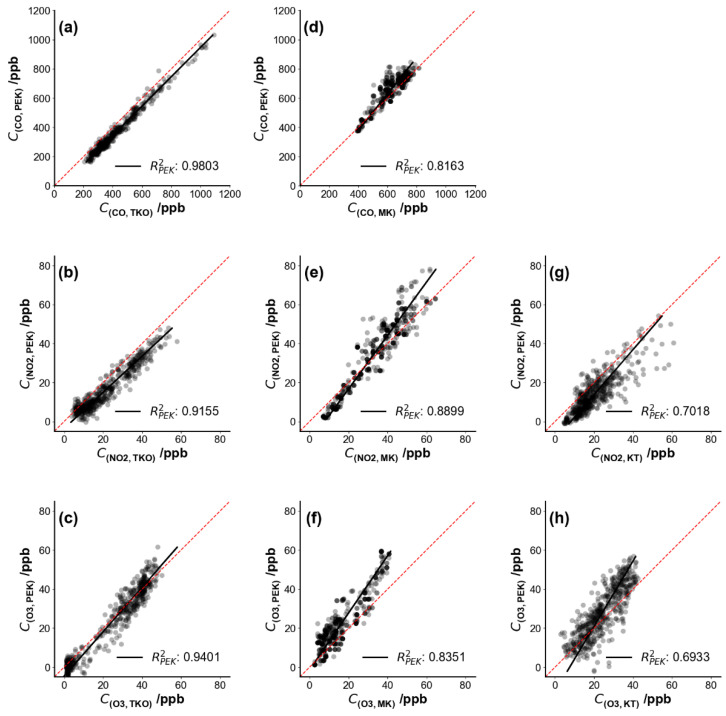
Scatter plots of gases measured by the Personal Exposure Kit (PEK) under laboratory calibration condition (Methodology 2.3.1 (I)) against concentration values collected by fixed monitors at the reference sites. Each sub-figure corresponds to the time series plots shown in Figure 6: (**a**–**c**) Tseung Kwan O (TKO), (**d**–**f**) Mong Kok (MK) and (**g**,**h**) Kwun Tong (KT) air quality monitoring sites. The dashed red line represents the identity line. Data resolution is set as 5 min.

Figure 6 shows pollutant concentrations as a time series over several days and Figure 7 shows the corresponding scatter plots of PEK concentrations against measured concentrations at the co-location sites TKO, MK and KT after the laboratory calibration procedures (Methodology 2.3.1 (I)). Carbon monoxide data from the KT site were not available. A brief PEK data loss occurred during the field testing period at the TKO site, so data from December 18 09:15 to December 18 11:45 did not form part of the analysis. Data below sensor detection limits during the cross-testing period were removed and then replaced using linear interpolation to obtain unknown values within a data set at the three co-location sites. Calibration parameters determined under laboratory conditions were applied across all field test periods. 

Figure 7 shows scatter plots for the three pollutant concentrations as measured by the PEK, plotted as a function of the concentrations at the co-located sites along with the coefficient of determination, R^2^. The measurements remain well-correlated even when the kit is moved from one site to another. According to a previous study [15], sensor correction algorithms often fail due to meteorological dependence when electrochemical sensors are deployed to a different place. However, the PEK measurements provided good quality data across multiple locations. On the basis of one-month sampling, no obvious sensor drift occurred to the PEK, and the experiment was a useful reference for protocol development on the frequency of calibration needed to maintain performance. Further studies may be designed on the quantification of sensor drift over much longer periods. 

#### 3.2.2. Sensor Change among Different Sites

**Figure 8:**
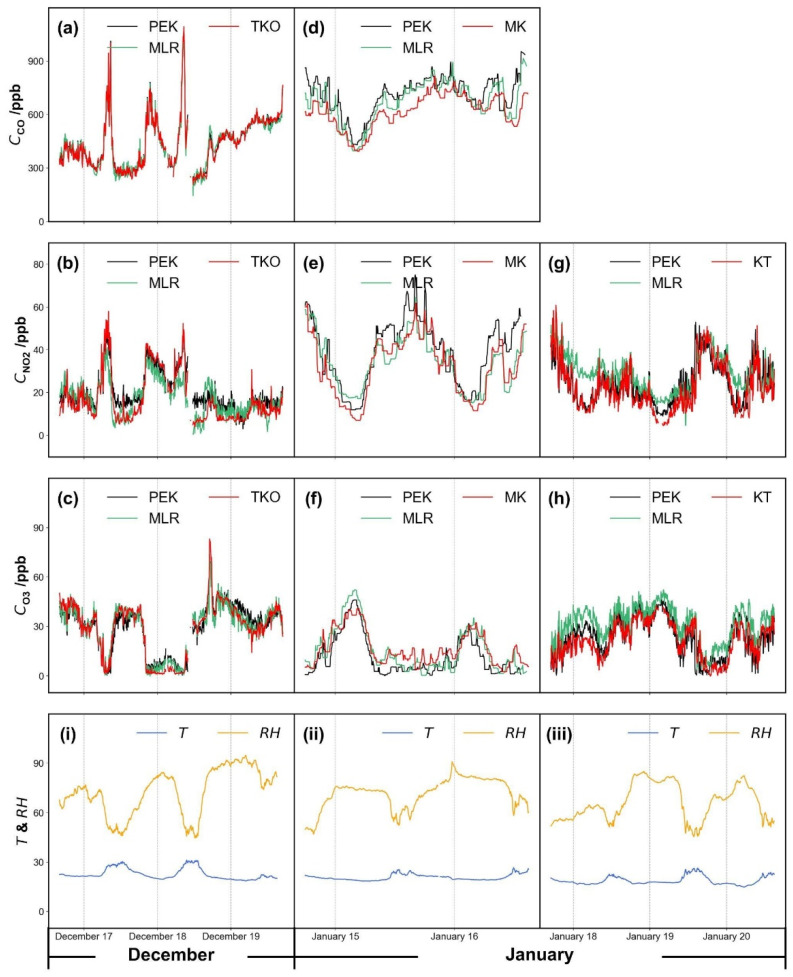
Time series plots of gases across the test period tuned via the multi-linear regression model compared with Personal Exposure Kit (PEK) measurements, which were calibrated following Protocol 2 at the Tseung Kwan O (TKO) site (**a**–**c**), at Mong Kok (MK) (**d**–**f**) and Kwun Tong (KT) (**g**,**h**) air quality monitoring sites under the same ambient conditions, including environmental factors, temperature (15 °C–30 °C) and humidity (45–90%) (**i**–**iii**). Data resolution is set as 5 min.

**Figure 9:**
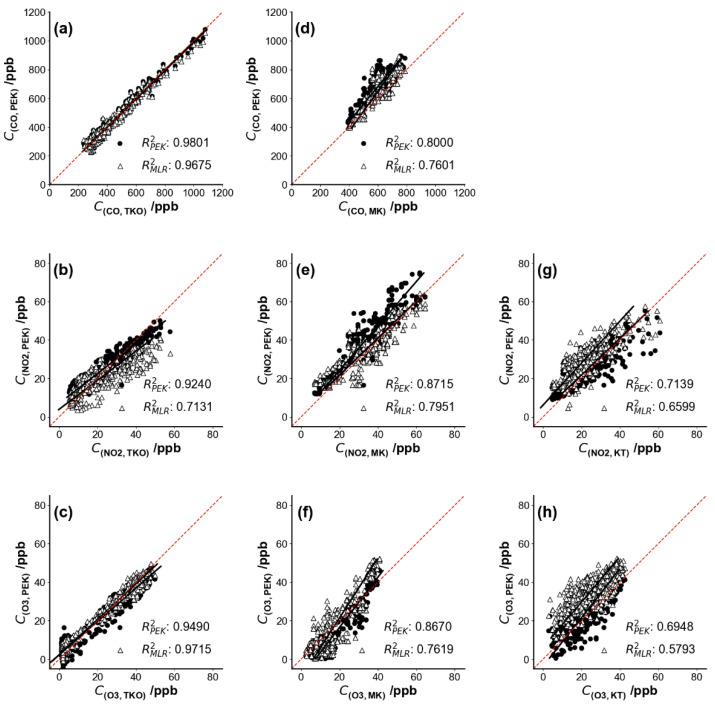
Scatter plots of gases measured by the Personal Exposure Kit (PEK) as PEK measurements under field co-location calibration condition (Methodology 2.3.1 (II)) against concentration values from fixed sites and R^2^ for each scenario: (**a**–**c**) Tseung Kwan O (TKO), (**d**–**f**) Mong Kok (MK) and (**g**,**h**) Kwun Tong (KT) air quality monitoring sites. Note: Black dots refer to PEK measurement and open triangles indicate raw sensor output tuned using multi-linear regression. Data resolution is set as 5 min.

PEK measurements were compared with raw sensor output corrected using multi-linear regression with parameters derived from co-location at the TKO site as described in Methodology 2.3.1 (II). Figure 8 compares tuned sensor output from TKO and the PEK measurements at the three reference sites. A narrow range of temperatures was encountered with a center point at ~25 °C. Correlation coefficients suggest good agreement between PEK measurements and reference data (shown in Figure 9). However, O_3_ measurements showed only fair correlation with fixed site data (R^2^_MLR_ = 0.56) after one month of operation. It is unclear what factors contributed to this limited agreement.

The PEK data is in better agreement than the multi-linear regression (MLR) adjusted data (across the period shown in Table 1) in terms of the mean absolute error (MAE), root mean square error (RMSE) and correlation coefficient (R^2^); see Table 1 for values. We defined the calibration procedures as Protocol 1 (Methodology 2.3.1 (I)), Protocol 2 (Methodology 2.3.1 (II)) and Method 1 for the multiple regression. 

**Figure 10:**
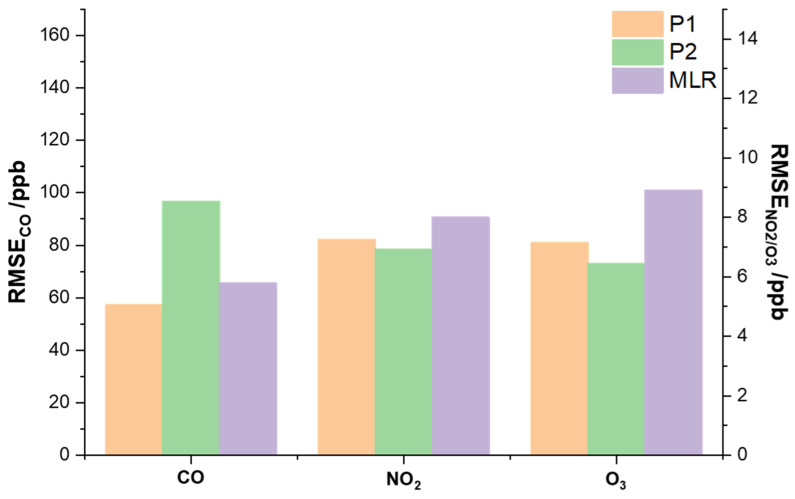
RMSE determined from respective calibration protocols along with the conventional multi-linear regression model: Protocol 1 (P1) refers to the Personal Exposure Kit (PEK) calibrated with standard gases under laboratory conditions; Protocol 2 (P2) refers to the PEK calibrated by collocation with government reference instruments and Method 1 (MLR) refers to raw sensor signal tuned by the multi-linear regression in the same circumstances.

The performance of the PEK at different reference sites in Hong Kong is shown in Table 1. Here we choose to examine RMSE as the evaluation criterion, instead of MAE, as MAE may not represent both the small and large errors which may appear in our study. RMSE can assess the discrepancies between the sample (reference pollutant data) and observed values (output from the kit). For each target gas, only residuals calculated during the validation period were included and residuals calculated during the calibration period were excluded. For example, under Protocol 1, residuals (or prediction errors) shown in Table 1 for the three sites excluded residuals calculated from the calibration period; similarly, for Protocol 2, residuals at MK and KT were included in calculations, but not TKO. Figure 10 shows RMSE comparison among the three calibration protocols, which averaged residuals of each pollutant at the stations and suggests that errors differ little. 

For CO, Protocol 1 is the best choice as a routine calibration protocol (*RMSE*_P1_ = 57.4 ppb), while Protocol 2 is less effective, with an RMSE 47.7% higher than under Method 1. NO_2_ shows approximately equal RSME values of ~8.0 ppb, and for O_3_, the highest RMSE is seen under Method 1 (*RMSE*_M1_ = 8.9 ppb).

Overall, these three protocols are largely in agreement for the three pollutants over a one-month observation period at different monitoring stations. The laboratory calibration (Protocol 1) proves robust for the PEK across the monitoring sites. However, if conventional instruments are not available for laboratory calibration, field co-location (Protocol 2) aids data quality assurance. Although multi-linear regression has acceptable RMSE values for CO, the discrepancies were larger for NO_2_ and O_3_ over longer periods (1 month). 

#### 3.2.3. PEK Output during a Simulated Personal Exposure Assessment

**Figure 11:**
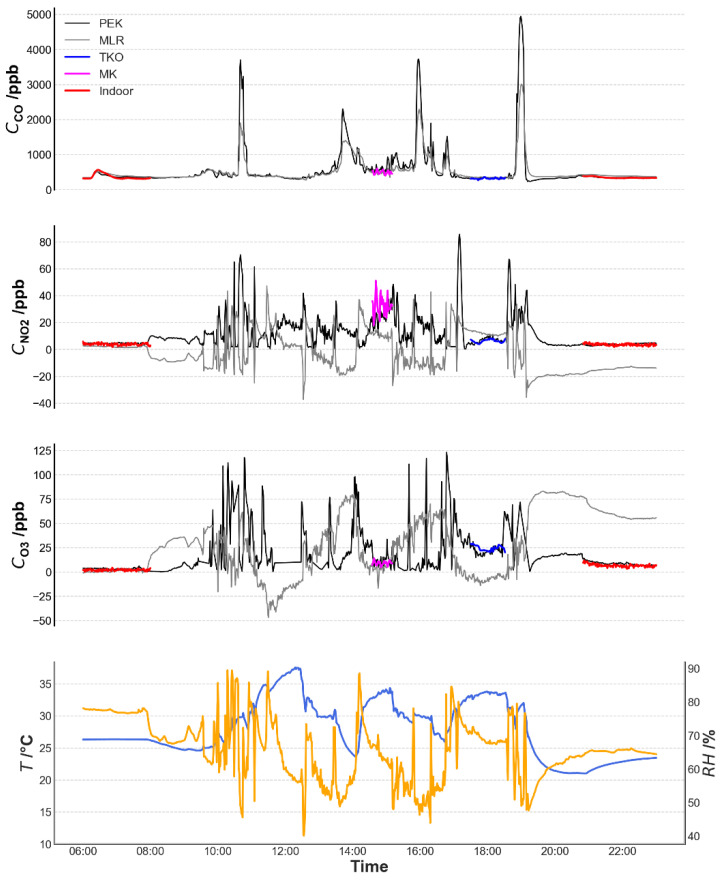
Temporal concentration variability of pollutants detected as Personal Exposure Kit (PEK) measurements and those tuned using the multi-linear regression model and ambient environmental factors as they vary simultaneously. Note: Data resolution is 1 min.

The kit was trialed as a personal exposure monitor across a range of urban microenvironments. It was carried on a backpack into various indoor and outdoor locations, which represent notable variations in pollutant concentrations. Figure 11 shows that the kit can capture short-period pollutant fluctuations even though temperature and humidity vary between the range of microenvironments. Corrections using the multi-linear regression model were not applicable to such unstable environments, especially for NO_2_ and O_3_ measurements. There is no obvious difference between the PEK measurements and the multi-linear regression model under the initial indoor conditions. However, the influence of environmental factors is problematic in output corrected using the multi-linear regression model. When the kit was taken back to the identical indoor conditions, both NO_2_ and O_3_ algorithms continued to fail. 

The agreement at roadside station MK is not as convincing as ambient station TKO, which may be caused by instantaneous vehicle emissions. Such emissions have very dynamic changes in concentration over short distances, and the distance between the sensors and the station air inlet due to space constraints may play a role. Smaller discrepancies were observed at TKO. This reinforces a frequent concern over the accuracy of measurement when deploying electrochemical sensors at disparate locations or at the same site at different times. 

Figure 11 shows that for CO, the general trend was identical except for the peaks. This suggests that the multi-linear regression model can be applied to different microenvironments for this pollutant (*r*_PEK,TKO_ = 0.804; *r*_MLR,TKO_ = 0.657; *r*_PEK,MK_ = 0.261; *r*_MLR,MK_ = 0.372). In general, the PEK is able to capture the short-period gaseous air pollutant concentrations experienced during personal exposure even when concentrations are highly variable. The multi-linear regression model is effective for CO transient concentration assessment but did not perform well for NO_2_ and O_3_ measurement in this case.

## 4. Conclusions and Future Work

The personal exposure kit demonstrated an excellent ability to provide output for O_3_, NO_2_ and CO concentration measurement that appears to be largely free of temperature-humidity effects in the laboratory and under field conditions. The PDF enabled dynamic baseline tracking method overcomes the sensitivity to ambient conditions often found with electrochemical cell-based systems. This is especially useful when the kit is used under conditions where temperature and humidity vary on diurnal and shorter time scales. Results show good linearity in controlled environments and a more stable NO_2_ signal response when used in indoor-to-outdoor applications. The performance of the dynamic baseline tracking exceeds that of the prevalent multi-linear regression model under the same conditions. In addition, comparison between PEK output and concentrations from the regression model in calibration protocols and changing environments indicates the dynamic baseline correction performs better, particularly when measuring NO_2_ and O_3_ at low ppb concentrations.

Additionally, validating the applicability of various calibration protocols, both in the laboratory and field, revealed that data from this kit shows acceptable results when deployed at a new site. Sensor-based monitors calibrated in the laboratory are not always easy to apply directly to field measurement, whereas the PEK calibrated system can provide reliable measurements in such cases. Our study shows that when standard gas calibration is not possible, co-located field calibration against regulatory monitors is valuable. Molecular diffusion delay via diffusion-based sampling impedes response times by seconds to a minute, so the capture of high-resolution measurements requires further work.

Overall, the dynamic baseline correction offers a promising technology that is of benefit for measurements made at many locations, especially pollutant hotspots that might best be assessed real-time. It will thus contribute to epidemiology and urban air quality characterization. The kit has been tested in a reasonable range of environments. However, further studies might also cover pollutant concentrations, temperature and humidity to establish its performance characteristics under a wider range of conditions. It is likely that the dynamic baseline correction could be applied to an advantage with other pollutants measured using microsensors sensitive to temperature and humidity conditions. It offers the potential for direct reading and on-site reporting of data of high quality without post-processing using data correction algorithms.

## Figures and Tables

**Table 1:** Statistical analysis of measurements from the Personal Exposure Kit (PEK) calibrated using Protocol 1 (P1), Protocol 2 (P2) and multi-linear regression (MLR) as mean absolute error (MAE), root mean square error (RMSE) and the coefficient of determination (R^2^) during the entire on-site test period at government monitoring stations Tseung Kwan O (TKO), Mong Kok (MK) and Kwun Tong (KT).

	**P1**			**P2**			**MLR**		
**CO**	**Validation**			**Calibration**	**Validation**		**Calibration**	**Validation**	
**ppb**	**TKO**	**MK**	**KT**	**TKO**	**MK**	**KT**	**TKO**	**MK**	**KT**
MAE	55.29	42.34	-	16.45	85.48	-	22.58	53.56	-
RMSE	58.88	55.93	-	21.66	96.74	-	28.07	65.51	-
R^2^	0.98	0.81	-	0.98	0.80	-	0.98	0.88	-
	**P1**			**P2**			**MLR**		
**NO_2_**	**Validation**			**Calibration**	**Validation**		**Calibration**	**Validation**	
**ppb**	**TKO**	**MK**	**KT**	**TKO**	**MK**	**KT**	**TKO**	**MK**	**KT**
MAE	5.44	5.63	6.93	4.16	6.76	4.26	5.83	5.16	8.59
RMSE	6.39	7.11	8.28	5.02	8.21	5.63	7.20	6.26	9.77
*R* ^2^	0.92	0.89	0.71	0.92	0.87	0.70	0.72	0.82	0.65
	**P1**			**P2**			**MLR**		
**O_3_**	**Validation**			**Calibration**	**Validation**		**Calibration**	**Validation**	
**ppb**	**TKO**	**MK**	**KT**	**TKO**	**MK**	**KT**	**TKO**	**MK**	**KT**
MAE	3.68	6.75	6.68	3.77	5.35	5.28	2.62	4.59	10.56
RMSE	4.76	8.37	8.33	4.44	6.48	6.42	3.19	5.73	12.07
*R* ^2^	0.94	0.81	0.70	0.95	0.87	0.67	0.97	0.86	0.56

## Data Availability

The data may be obtained on request from the corresponding author.

## References

[1] Jantunen E. (2002). A summary of methods applied to tool condition monitoring in drilling. Int. J. Mach. Tools Manuf..

[2] Monn C. (2001). Exposure assessment of air pollutants: A review on spatial heterogeneity and indoor/outdoor/personal exposure to suspended particulate matter, nitrogen dioxide and ozone. Atmos. Environ..

[3] Sexton M., Hebel J.R. (1984). A clinical trial of change in maternal smoking and its effect on birth weight. JAMA.

[4] (2016). World Health Organization Ambient air pollution: A global assessment of exposure and burden of disease. Clean Air J..

[5] Kelly F.J., Fussell J.C. (2015). Air pollution and public health: Emerging hazards and improved understanding of risk. Environ. Geochem. Heal..

[6] Beeson W.L., E Abbey D., Knutsen S.F. (1998). Long-term concentrations of ambient air pollutants and incident lung cancer in California adults: Results from the AHSMOG study.Adventist Health Study on Smog. Environ. Heal. Perspect..

[7] Dockery D.W., Pope C.A. (1994). Acute respiratory effects of particulate air pollution. Annu. Rev. Public Health.

[8] Katsouyanni K., Touloumi G., Spix C., Schwartz J., Balducci F., Medina S., Rossi G., Wojtyniak B., Sunyer J., Bacharova L. (1997). Short term effects of ambient sulphur dioxide and particulate matter on mortality in 12 European cities: Results from time series data from the APHEA project. BMJ.

[9] Baron R., Saffell J. (2017). Amperometric Gas Sensors as a Low Cost Emerging Technology Platform for Air Quality Monitoring Applications: A Review. ACS Sens..

[10] Che W., Frey H.C., Fung J.C., Ning Z., Qu H., Lo H.K., Chen L., Wong T.-W., Wong M.K., Lee O.C. (2020). PRAISE-HK: A personalized real-time air quality informatics system for citizen participation in exposure and health risk management. Sustain. Cities Soc..

[11] Mead M., Popoola O., Stewart G., Landshoff P., Calleja M., Hayes M., Baldovi J., McLeod M., Hodgson T., Dicks J. (2013). The use of electrochemical sensors for monitoring urban air quality in low-cost, high-density networks. Atmos. Environ..

[12] Pang X., Shaw M.D., Lewis A.C., Carpenter L.J., Batchellier T. (2017). Electrochemical ozone sensors: A miniaturised alternative for ozone measurements in laboratory experiments and air-quality monitoring. Sens. Actuators B Chem..

[13] Pang X., Shaw M.D., Gillot S., Lewis A.C. (2018). The impacts of water vapour and co-pollutants on the performance of electrochemical gas sensors used for air quality monitoring. Sens. Actuators B Chem..

[14] Wei P., Ning Z., Ye S., Sun L., Yang F., Wong K.C., Westerdahl D., Louie P.K.K. (2018). Impact Analysis of Temperature and Humidity Conditions on Electrochemical Sensor Response in Ambient Air Quality Monitoring. Sensors.

[15] Spinelle L., Gerboles M., Villani M.G., Aleixandre M., Bonavitacola F. (2017). Field calibration of a cluster of low-cost commercially available sensors for air quality monitoring. Part B: NO, CO and CO_2_. Sens. Actuators B Chem..

[16] Topalovic D., Davidovic M., Jovanović M., Bartonova A., Ristovski Z., Jovašević-Stojanović M. (2019). In search of an optimal in-field calibration method of low-cost gas sensors for ambient air pollutants: Comparison of linear, multilinear and artificial neural network approaches. Atmos. Environ..

[17] Popoola O., Stewart G., Mead I., Jones R. (2017). Development of a baseline-temperature correction methodology for electrochemical sensors and its implications for long-term stability. Atmos. Environ.

[18] Sun L., Westerdahl D., Ning Z. (2017). Development and Evaluation of a Novel and Cost-Effective Approach for Low-Cost NO_2_ Sensor Drift Correction. Sensors.

[19] Sohn J.H., Atzeni M., Zeller L., Pioggia G. (2008). Characterisation of humidity dependence of a metal oxide semiconductor sensor array using partial least squares. Sens. Actuators B Chem..

[20] Wang Y., Li J., Jing H., Zhang Q., Jiang J., Biswas P. (2015). Laboratory Evaluation and Calibration of Three Low-Cost Particle Sensors for Particulate Matter Measurement. Aerosol Sci. Technol..

[21] Mijling B., Jiang Q., De Jonge D., Bocconi S. (2018). Field calibration of electrochemical NO2 sensors in a citizen science context. Atmos. Meas. Tech..

[22] Carslaw N. (2007). A new detailed chemical model for indoor air pollution. Atmos. Environ..

[23] Warburton P.R., Pagano M.P., Hoover R., Logman A.M., Crytzer K., Warburton Y.J. (1998). Amperometric Gas Sensor Response Times. Anal. Chem..

[24] Piedrahita R., Xiang Y., Masson N., E Ortega J.K., Collier A., Jiang Y., Li K., Dick R.P., Lv Q., Hannigan M. (2014). The next generation of low-cost personal air quality sensors for quantitative exposure monitoring. Atmos. Meas. Tech..

[25] Malings C., Tanzer R., Hauryliuk A., Kumar S.P.N., Zimmerman N., Kara L.B., Presto A.A., Subramanian R. (2019). Development of a general calibration model and long-term performance evaluation of low-cost sensors for air pollutant gas monitoring. Atmos. Meas. Tech..

[26] Spinelle L., Gerboles M., Villani M.G., Aleixandre M., Bonavitacola F. Calibration of a cluster of low-cost sensors for the measurement of air pollution in ambient air. Proceedings of the IEEE SENSORS 2014.

[27] McKinney W. (2012). Python for Data Analysis: Data Wrangling with Pandas, NumPy, and IPython.

[28] Brantley H.L., Hagler G.S.W., Kimbrough E.S., Williams R.W., Mukerjee S., Neas L.M. (2014). Mobile air monitoring data-processing strategies and effects on spatial air pollution trends. Atmos. Meas. Tech..

[29] Shairsingh K.K., Jeong C.-H., Wang J.M., Evans G.J. (2018). Characterizing the spatial variability of local and background concentration signals for air pollution at the neighbourhood scale. Atmos. Environ..

